# The antioxidant N-acetylcysteine prevents cortical neuropathological phenotypes caused by adolescent Δ-9-tetrahydrocannabinol exposure in male rats

**DOI:** 10.1038/s41398-025-03580-4

**Published:** 2025-10-06

**Authors:** Hanna J. Szkudlarek, Rajkamalpreet Singh Mann, Krystyna Wieczerzak, Mohammed Halit Sarikahya, Taygun C. Uzuneser, Marta De Felice, Mar Rodríguez-Ruiz, Juan Pablo Galindo, Mathusha Pusparajah, Shawn N. Whitehead, Walter J. Rushlow, Daniel B. Hardy, Susanne Schmid, Ken K.-C. Yeung, Steven R. Laviolette

**Affiliations:** 1 Addiction Research Group, University of Western Ontario, London, ON Canada; 2 Department of Anatomy and Cell Biology, University of Western Ontario, London, ON Canada; 3 Department of Chemistry, University of Western Ontario, London, ON Canada; 4 Department of Clinical Neurological Science, University of Western Ontario, London, ON Canada; 5 Department of Physiology and Pharmacology; Schulich School of Medicine & Dentistry; University of Western Ontario, London, ON Canada; 6 Lawson Health Research Institute, London, ON Canada; 7 Department of Psychiatry, University of Western Ontario, London, ON Canada; 8 Children’s Health Research Institute, London, ON Canada

**Keywords:** Neuroscience, Physiology

## Abstract

Clinical and pre-clinical evidence demonstrates that adolescent Δ-9-tetrahydrocannabinol (THC) exposure, the primary psychoactive component of cannabis, increases the risk of developing neuropsychiatric symptoms in later life. The medial prefrontal cortex (mPFC) serves as a pathophysiological nexus point underlying many cannabis-related pathophysiological outcomes. Nevertheless, the molecular mechanisms underlying these risk factors are poorly understood. THC increases oxidative stress, which is a well-established causal factor for increased neuropsychiatric risk, including schizophrenia. N-acetylcysteine (NAC) is an antioxidant glutathione precursor that normalizes glutamate and GABA activity in neuropathological states. We examined if NAC may prevent the pathophysiological impacts of THC using a rodent model of adolescent brain development and chronic THC exposure. We report that NAC treatment prevents cognitive, synaptic, neuronal and neurochemical deficits induced by adolescent THC. These findings highlight the critical role of THC-induced oxidative stress as a contributing factor to cannabinoid-mediated neuropsychiatric risk and identifies a novel antioxidant treatment candidate for the prevention and/or reversal of these pathophysiological outcomes.

## Introduction

Adolescent exposure to high potency cannabis is a significant risk factor for the later emergence of schizophrenia [1–3]. The mechanisms underlying the association between adolescent cannabis use and risk of psychotic and cognitive symptoms later in life have been explored in various animal models using exposure to escalating doses of Δ-9-tetrahydrocannabinol (THC), the primary psychoactive compound in cannabis. These studies have revealed that adolescent THC exposure induces core schizophrenia-related endophenotypes resembling positive and negative symptoms, such asocial motivation deficits, affective dysregulation, cognitive and sensorimotor gating abnormalities [4, 5]. They have also identified a plethora of pathological molecular and neuronal phenotypes observed in schizophrenia patients, confirming the translational significance of these pre-clinical models [6, 7]. Importantly, high-potency cannabis products and purified THC extracts are increasingly consumed during adolescence, a period of profound neurodevelopmental vulnerability [8, 9]. Thus, there is an urgent need to characterize the specific neuropathological mechanisms underlying the impacts of adolescent THC exposure on neuropsychiatric risk and to identify potential intervention strategies aimed at preventing or reversing these outcomes [10, 11].

The neurodevelopmental sequelae of psychiatric disorders involve intersecting genetic [12, 13] and environmental factors [14]. Environmental insults that trigger neuroinflammatory responses during neurodevelopment, e.g. early life stress or chronic drug exposure [15–17], may lead to redox dysfunction and chronic oxidative stress, strongly linked to the pathophysiology of schizophrenia [18–22]. The endocannabinoid system plays a crucial regulatory role during neurodevelopment [23–27], and disruptions to this system via exposure to extrinsic cannabinoids have well-established neuropathological consequences [28]. The main cerebral target for THC is type-1 cannabinoid receptors (CB1R), which are widely expressed in the brain and regulate various functions including neurotransmitter release, neuro-astroglial communication, and cellular bioenergetic processes [29–32]. Indeed, CB1R function is a crucial player in central inflammatory and redox phenomena, underscoring the potential impact of neurodevelopmental THC exposure on these neurophysiological functions. Although THC possesses neuroprotective antioxidant properties through non-CB1R pathways [33], it also upregulates plasma inflammatory cytokines [34], increases vulnerability to ischemic stroke in young people [35], increases mitochondrial dysfunction [36, 37] and alters gene pathways associated with mitochondrial oxidative phosphorylation [38]. Unlike endogenous cannabinoids, THC also increases mitochondrial hydrogen peroxide production [39], and reactive oxygen species (ROS) production and neuronal apoptosis in mPFC [32], a brain region critically involved in neuropsychiatric pathology. Exposing cerebral mitochondria to THC was reported to induce a ten-fold increase in free radical leak and increased H_2_O_2_ production [40], while in epithelial cells, THC upregulated inflammation-related genes and downregulated antioxidant-related genes [34]. Collectively, this data strongly suggests that prolonged THC exposure can induce significant oxidative stress in neuronal populations and may thus dysregulate neurodevelopmental processes required for healthy cognitive and affective function.

Treatments counteracting oxidative stress, such as the antioxidant, N-acetylcysteine (NAC), have proven effective in preventing the emergence of behavioural and cognitive disruptions in various models of neurodevelopmental disorders [10, 41–43]. Here, we hypothesized that NAC treatment may prevent THC-induced dysregulations in central redox signaling pathways linked to schizophrenia-related endophenotypes and mitigate impairments in social behaviour, cognitive deficits and sensorimotor gating, along with underlying aberrations in relevant neurotransmitter systems and electrophysiological parameters. We report that NAC effectively prevents the harmful effects of adolescent THC exposure, highlighting its potential as a therapeutic intervention for treating long-term neuropsychiatric consequences of chronic developmental cannabis exposure. Our findings also underscore the crucial role of central redox mechanisms, particularly in the mPFC, in mediating the pathophysiological effects of THC.

## Materials and methods

### Animals

Male Sprague Dawley rats (total n = 133; 4 independent cohorts) (Charles River; Quebec, Canada) arrived at postnatal day (PND) 28. Rats were group housed (2–3 rats per cage) at standard conditions (temperature: +24 ± 2 °C; humidity: 55 ± 10%; light schedule: 12:12 h, light on at 7AM) with food and water available *ad libitum* unless stated otherwise. Rats were assigned to experimental groups randomly at the beginning of adolescent treatment. All procedures were approved by the Institutional Animal Care Committee and complied with the Canadian Council on Animal Care guidelines.

### Drugs and treatments

**Fig. 1:**
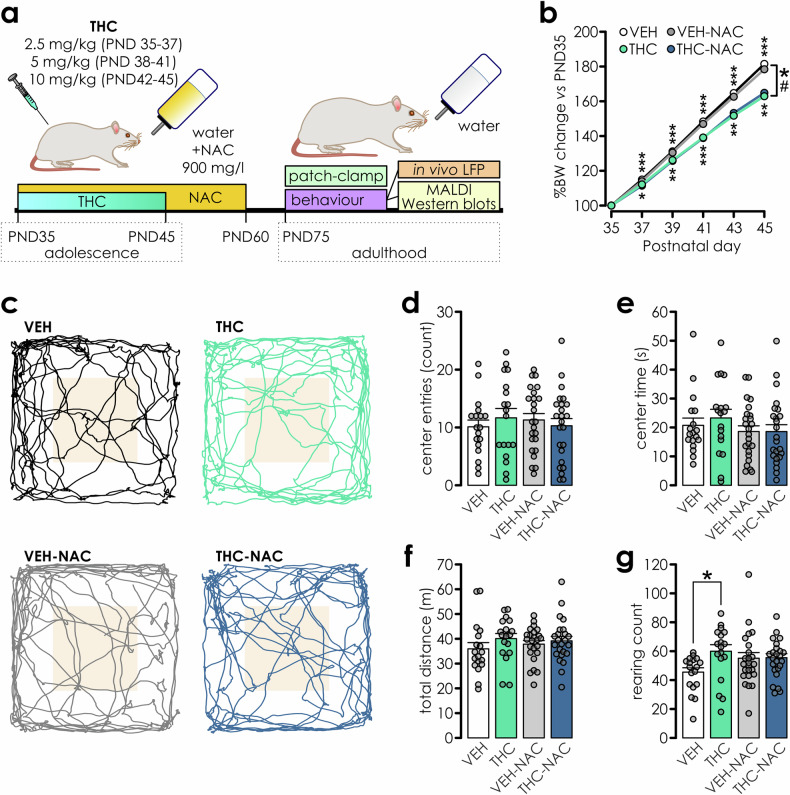
Experimental time-line and effects of adolescent treatment on body weight, anxiety, motility and stereotypy behaviour. **a** Schematic illustration for experimental treatment timelines. **b** Acute effects of adolescent THC exposure on body weight are not impacted by NAC co-treatment (n: VEH = 38; THC = 40; VEH-NAC = 24; THC-NAC = 24). Repeated measures two-way ANOVA: PND day: F_(4,488)_ = 2301.214, p < 0.001; PND day*IP treatment: F_(4,488)_ = 29.413, p < 0.001; PND day*oral treatment: F_(4,488)_ = 0.07, p = 0.991; PND day*IP*oral treatment: F_(4,488)_ = 1.169, p = 0.324; pairwise comparisons VEH *vs*. THC at PND37-45: p < 0.001; VEH-NAC *vs*. THC-NAC at PND37: p = 0.014, PND39: p = 0.003, PND41: p < 0.001, PND43: p = 0.003, PND47: p = 0.001). **c** Example motor activity traces for each treatment group with the center area highlighted with pale orange background. Rats in all treatment groups (n: VEH = 18, THC = 18, VEH-NAC = 24; THC-NAC = 24) entered the center of the arena with similar frequency **d** and duration **e**. Although there were no **d**ifferences betwe**e**n groups in total activity levels **f**, THC treatment significantly increased stereotyped rearing frequency **g** (two-way ANOVA: IP treatment: F_(1,80)_ = 4.19, p = 0.044; oral treatment: F_(1,80)_ = 0.509, p = 0.47; IP*oral treatment: F_(1,80)_ = 3.77, p = 0.056; pairwise comparisons: VEH *vs*. THC: p = 0.01). Significance indicators on the right point significance level for main effects observed between VEH *vs*. THC (*) and VEH-NAC *vs*. THC-NAC (#). Data are presented as mean± S.E.M. with individual data points superimposed on bar graphs, for this and all subsequent figures. Asterisks = *p < 0.05, **p < 0.01, ***p < 0.001 for this and all subsequent figures.

Adolescent THC treatment was conducted between PND35 and PND45 (Fig. 1a). Rats were treated with increasing doses of THC as described previously [5]: 2.5 mg/kg (PND35-37), 5 mg/kg (PND38-41) and 10 mg/kg (PND42-45) or with vehicle. For NAC intervention experiments, rats were co-treated with N-acetylcysteine (NAC, Sigma Aldrich; Cat#A7250) administered *ad libitum* in drinking water (900 mg/l; PND35-60) and fresh solution was prepared every 2 days. Cremophor EL (Sigma Aldrich) was added to THC-EtOH solution (1 g/100 ml EtOH; Cayman Chemicals), vortexed and EtOH was evaporated using nitrogen stream. Subsequently, the THC solution was diluted in physiological saline to a final concentration of 2.6 mg/ml and 5% Cremophor. Vehicle contained 5% Cremophor in saline.

### Behavioural experiments

All behavioural experiments were conducted on adult animals >PND75, video recorded and analyzed offline using Behaview software (www.pmbogusz.net) and Any-maze (San Diego Instruments, RRID:SCR_014289) software. Experiments were analyzed in a blinded manner and all behavioural tests were independently replicated at least once. Sample sizes per experimental group were chosen based on previously reported similar studies [4, 5].

#### Open field test (OF)

The Open field apparatus was a square arena (80 × 80, 50 cm high wall) made of black acrylic and brightly illuminated. Naïve rats were placed in the middle of the OF and explored it freely for 10 min. The first five min of the test was used to measure: number of entries into the center zone (40 × 40 cm in the middle of OF) and center zone time. Locomotor functions were assessed by measuring total distance travelled. Stereotypy was analyzed from rearing counts and duration.

#### Social interaction test (SI)

**Fig. 2:**
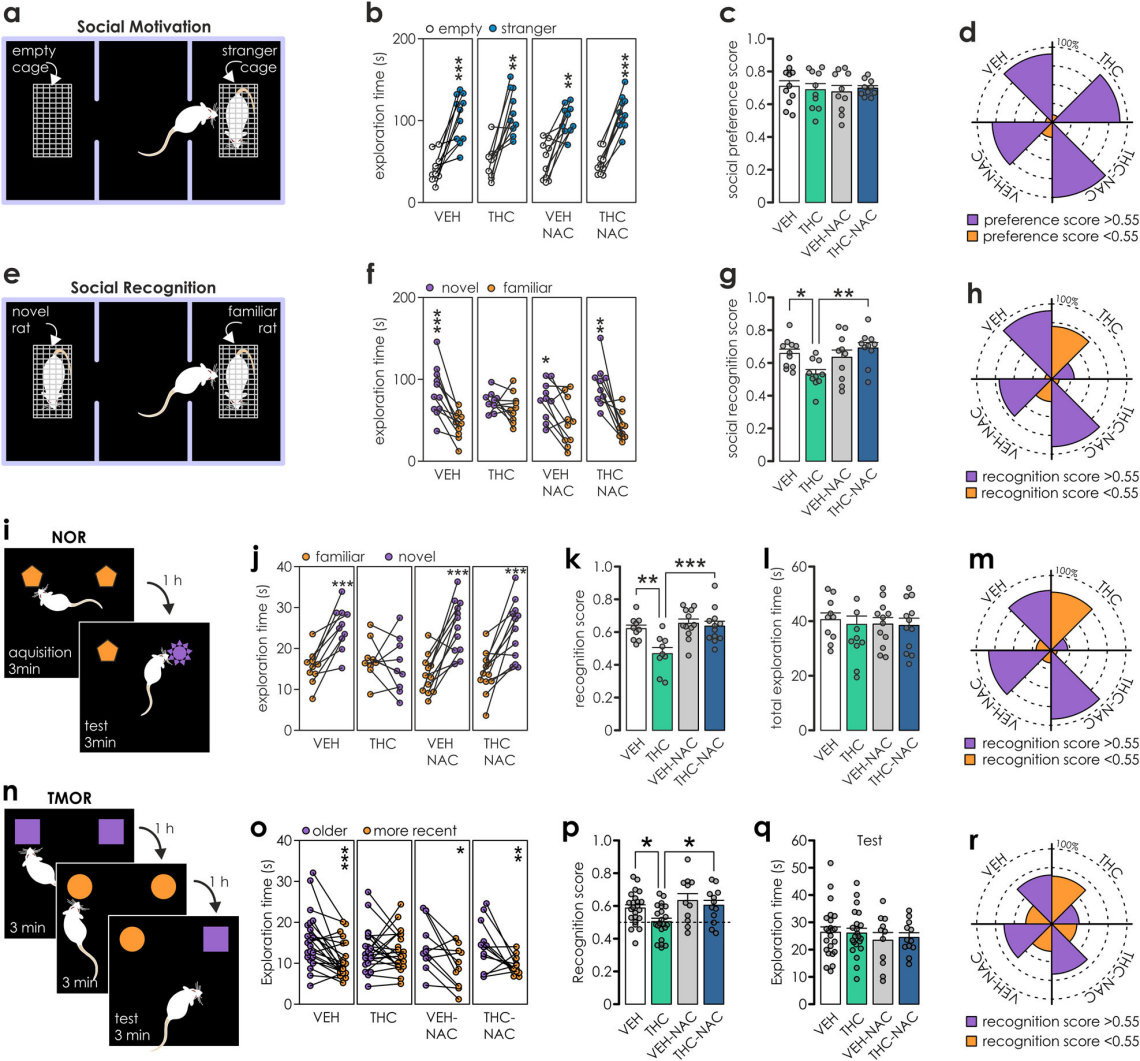
NAC prevents sociability impairments induced by adolescent exposure to THC. Social interaction test (n:VEH = 11, THC = 10, VEH-NAC = 10; THC-NAC = 10). Schematic of the three-chamber test and procedure for social motivation **a** and social recognition **e** experimental phases. **b** Duration that rats spent exploring an empty cage *vs*. a cage containing stranger rat. Paired *t*-tests revealed that rats in all experimental groups preferred to explore the stranger rat (VEH: t_(10)_ = −5.55, p < 0.001; THC: t_(9)_ = −4.69, p = 0.001; VEH-NAC: t_(9)_ = −4.67, p = 0.001; THC-NAC: t_(9)_ = −12.15, p < 0.001). **c** Social preference s**c**ores were similar across all treatment groups (two-way ANOVA: IP treatment: F_(1,37)_ = 0.001, p = 0.981; oral treatment: F_(1,37)_ = 0.139, p = 0.712; IP*oral treatment: F_(1,37)_ = 0.423, p = 0.52). **d** Radial plot depicting the percentage of animals displaying preference toward stranger rat (score > 0.55; purple). Note no differences between groups. **f** Duration that rats spent exploring a novel *vs*. familiar rat. Paired *t*-tests: VEH: t_(10)_ = −5.464, p < 0.001; THC: t_(9)_ = −1.036, p = 0.327; VEH-NAC: t_(9)_ = −3.121, p = 0.012; THC-NAC: t_(9)_ = −4.65, p = 0.001. **g** Social recognition scores were lower for THC-exposed rats (two-way ANOVA: IP treatment: F_(1,37)_ = 1.058, p = 0.31; oral treatment: F_(1,37)_ = 4.078, p = 0.051; IP*oral treatment: F_(1,37)_ = 7.43, p = 0.01, pairwise comparisons: VEH *vs*. THC p = 0.011; THC *vs*. THC-NAC p = 0.002). **h** Radial plot depicting the percentage of animals displaying preference towards a novel rat (score > 0.55; purple). Note the inverted pattern in THC, but not in the THC-NAC group. *p < 0.05, **p < 0.01. **i** Schematic summary of the novel object recognition (NOR) procedure. **j** Only THC-treated rats failed to recognize novel objects (n: VEH = 10, THC = 9, VEH-NAC = 12; THC-NAC = 12). Paired *t*-tests for familiar *vs*. novel object: VEH: t_(9)_ = −5.861, p < 0.001; THC: t_(8)_ = 0.603, p = 0.563; VEH-NAC: t_(11)_ = −5.446, p < 0.001; THC-NAC: t_(11)_ = −4.465, p < 0.001. **k** Recognition memory was significantly impaired only in the THC-exposure group. Two-way ANOVA: IP treatment F_(1,39)_ = 8.309, p = 0.006; oral treatment: F_(1,39)_ = 11.601, p = 0.002 and IP*oral treatment: F_(1,39)_ = 5.291, p = 0.027; pairwise comparisons: THC *vs*. VEH: p = 0.001; THC-NAC *vs*. VEH: p = 0.664; THC *vs*. THC-NAC: p < 0.001. **l** No differences in total exploration time were observed across groups. Two-way ANOVA: IP treatment: F_(1,39)_ = 0.001, p = 0.97; oral treatment: F_(1,39)_ = 0.626, p = 0.433; IP*oral treatment: F_(1,39)_ = 0.282, p = 0.599. **m** Percentage of rats displaying novelty recognition score below 0.55 was higher only in the THC group, but not in NAC treated groups. **n** Schematic of the object recognition in temporal order (n: VEH = 23, THC = 22, VEH-NAC = 11; THC-NAC = 12). **o** Only the THC rats did not prefer the older object. Paired *t*-tests: VEH: t_(22)_ = 3.986, p < 0.001; THC: t_(21)_ = 0.25, p = 0.805; NAC: t_(10)_ = 3.123, p = 0.011; THC-NAC: t_(11)_ = 3.364, p = 0.006. **p** Recognition score was lower in THC rats. Two-way ANOVA: IP treatment: F_(1,64)_ = 4.507; p = 0.038; oral treatment: F_(1,64)_ = 7.435, p = 0.008; IP*oral treatment: F_(1,64)_ = 0.747, p = 0.391; pairwise comparisons: THC *vs*. VEH: p = 0.013; THC *vs*. THC-NAC: p = 0.039. **q** All rats displayed similar exploration times. Two-way ANOVA: IP treatment: F_(1,64)_ = 0.028; p = 0.869; oral treatment: F_(1,64)_ = 1.047, p = 0.31; IP*oral treatment: F_(1,64)_ = 0.088, p = 0.768. **r** Radial plot capturing the inverted percentage of animals with impaired recency recognition in THC group (score < 0.55).

The social interaction apparatus consisted of a transparent acrylic arena divided into three equally sized chambers connected with guillotine doors. First, rats were habituated to the arena (5 min center chamber +8 min entire apparatus). The next day rats underwent (Fig. 2a, e): social motivation test (phase 1) and the social recognition test (phase2). First, a rat was placed in the center chamber (guillotine doors in place) for 5 min. Next, two wire cages (one empty and one with a stranger rat) were positioned in the side chambers, the guillotine doors were removed, and the test rat was allowed to explore for 8 min. Finally, a novel unfamiliar rat was introduced to the previously empty wire cage and the test rat explored the apparatus for 8 min. The exploration of the wire enclosures (sniffing the cage) was measured. The sociability scores were calculated as: social motivation score = exploration of stranger/(total exploration in phase 1) and social recognition score = exploration of novel rat/(total exploration in phase 2).

#### Object recognition tests

The tests were conducted in the OF apparatus using different sets of objects and were preceded with habituation (10 min) to the OF a day before. Objects and apparatus were cleaned with 70% EtOH between the tests and rats. The position of odd, novel and recent object was counterbalanced between rats. Animals that did not explore one of the objects at any stage of the test or toppled any of the objects during the procedure, were excluded from the analysis.

#### Simultaneous oddity discrimination task (SOD)

The test consisted of a single phase (5 min; Suppl Fig. 1a) and rats were presented with three objects, two matching and one odd. The oddity discrimination Score=time exploring the odd object/total exploration time.

#### Novel object recognition (NOR)

The test consisted of a learning and recognition phase (3 min each; separated by 1 h; Fig. 2i). In phase-1, rats were presented with two identical objects, while in phase-2 one of the objects was novel. Object exploration times (sniffing of the object) were used to calculate the recognition score = time exploring novel object/time exploring both objects.

#### Object recognition in temporal order (TMOR)

The test consisted of: learning phase-1 and −2, and the recognition phase-3 (3 min each, separated by 1 h; Fig. 2n). During learning phases rats were presented with two identical objects (one pair/phase). On the recognition phase one object from each learning pair was presented. Object exploration times were used to calculate the recognition score = time exploring older object/time exploring both objects at phase-3.

#### Set shifting and reversal learning

Rats were food restricted (15 g food/day) until they reached 85% of their initial body weight and habituated to a food reward (45 mg sucrose pellets; BioServ, USA). A sound attenuated box (Med-Associates, St Albans VT, USA) equipped with two levers, cue lights above the levers and food cup connected to a pellet dispenser and controlled with customized software procedures (MED-PC IV, Med-Associates) was used for testing detailed in Supplementary Materials & Methods.

#### Acoustic startle responses (ASRs)

The assessment of acoustic reactivity, sensory filtering and sensorimotor gating was conducted in sound-attenuated startle boxes (LE116; Panlab) using the StartFear system (Panlab; Cat#76-0002 & Cat#76-0702) and STARTLE software module (PACKWIN-CSST, PACKWIN version 2.0; Panlab). Rats were placed in plastic startle tubes and positioned on a weight transducing platform enclosed in a sound-attenuating chamber. Prior to the behavioural procedures involving auditory startle responses (ASRs), rats were handled and acclimated (5 min) to the startle boxes and background noise (65 dB white noise). Next, to assess basal startle responding, animals were exposed to startle tones of increasing intensity (65–115, 5 dB increment, 60 s interval). The following day the prepulse-inhibition of startle response was assessed by exposing rats to a startle stimulus (105 dB for 20 ms) preceded (100 ms) by a pre-pulse (77, 80 and 83 dB for 10 ms) repeated 10 times for each prepulse intensity at pseudo-random interval (15–20 s, average of 17.5 s). Finally, short term habituation was measured by exposing rats to 50 startle pulses (110 dB, 15 s interval). Habituation score was calculated by dividing the median value of the last ten startle responses by the median of first three responses.

### In vivo electrophysiology

Following behavioural experiments, animals were implanted with extracellular recordings electrodes under deep ketamine-xylazine anaesthesia (i.p.; ketamine: 80 mg/kg, Vetoquinol; xylazine: 6 mg/kg, Bayer) using a sterile stereotaxic procedure. Electrodes were made of polyimide coated stainless-steel wire (120 μm; WPI) secured in the electrode pedestal (MS636, PlasticsOne) and targeted mPFC: +3.2 mm anterior and −0.8 mm lateral from Bregma, and 3.2 mm ventral to cortical surface. A stainless-steel bone screw was implanted above the cerebellum and served as the grounding and reference electrode. The implant was secured in place with 3 additional bone screws and dental cement. Rats were recovering for 7 days before recordings. Local field potential signals (LFP) were acquired at a 1 kHz rate using RZ6 processor (TDT) and band-pass filtered between 0.5–300 Hz. Data were analyzed using custom made scripts in Matlab.

### Patch-clamp electrophysiology

Patch-clamp experimental procedures are detailed in the Supplementary Materials and Methods. Briefly, adult rats (PND75-PND130) with histories of adolescent THC *vs*. VEH treatments were anesthetised with isoflurane and decapitated (n = 5–8 animals/group). Brains were quickly extracted and sliced (300 μm) for whole cell recordings in current-clamp and voltage-clamp mode.

### Matrix assisted laser desorption/ionization mass spectrometry (MALDI-MS)

Rats were given an overdose of pentobarbital (Euthanyl; 270 mg/ml), and brains were removed and flash frozen (–80 °C). For quantification of multiple neurotransmitters and metabolites two different matrices were used, zinc oxide nanoparticles (ZnO-NP, Sigma-Aldrich, St. Louis, MO) and 4-(Anthracen-9-yl)-2-fluoro-1-methylpyridin-1-ium iodide (FMP-10, TAG-ON, Uppsala, Sweden; Cat#T1001) and are detailed in the Supplementary Materials and Methods.

### Statistics

All data are presented as mean ± standard error of the mean (S.E.M.) and were analyzed using SPSS software (IBM; RRID:SCR_016479) unless stated otherwise. Normally *vs*. non-normally distributed data (Kolmogorov-Smirnoff test; p < 0.05) were tested using appropriate parametric *vs*. non-parametric analysis followed with post-hoc tests, where appropriate. The data sets were tested with either two-way ANOVA or repeated measures two-way ANOVA with adolescent IP treatment and oral treatment set as fixed factors and repeated measures set to the following: postnatal day (for detecting body weight changes during the treatment), pulse intensity (for assessing startle reactivity), pre-pulse intensity (for assessing sensory-motor gating in PPI test) and injected current amplitude (for detecting excitability changes in patch-clamp experiment). ANOVAs were followed with Sidak-corrected, post-hoc pairwise comparisons. Two-tailed paired *t*-tests were used to detect within-group differences between exploration times in social behaviour, object recognition tests and habituation of the startle response.

## Results

Male adolescent rats were subdivided into four treatment groups undergoing either chronic exposure to escalating doses of THC or VEH with either oral co-treatment with N-acetylcysteine (NAC; Fig. 1a) or plain water. Consistent with previous studies [44], THC-exposure modestly reduced weight gain during the exposure period (Fig. 1b) and NAC co-treatment did not counteract this effect.

### Effects of adolescent THC exposure on motility, stereotypic and anxiety-like behaviours

Adolescent THC exposure induces anxiogenic phenotypes and increases stereotypy behaviours in the open field task at adulthood [4, 5, 10]. Stereotypy is commonly observed in schizophrenia, characterized by repetitive, perseverative motor behaviours associated with negative symptom clusters [45]. We found that no treatment conditions affected locomotion or thigmotaxis measured in the OF test (Fig. 1c–f), however THC rats displayed increased stereotypy with significantly increased rearing counts *vs*. controls. NAC co-treatment prevented this THC effect (Fig. 1g), though rearing counts in both NAC treatment groups were elevated compared to VEH controls. Importantly, a recent study has shown that NAC treatment was not able to prevent THC-induced impairments in anxiety [46], suggesting that NAC might be less efficient in mitigating affective impairments that rely on amygdala function.

### NAC treatment prevents THC-induced impairments in social behaviours

Adolescent THC exposure impairs social behaviours at adulthood [4, 5, 10] and clinically, such deficits are well-established ‘negative’ symptom endophenotypes of schizophrenia. We used a social motivation/memory test to determine if NAC may prevent these deficits (Fig. 2a, e). Baseline sociability was similar across all cohorts with rats preferring to explore novel rat *vs*. empty enclosures (Fig. 2b). Social preference scores were uniform across treatments (Fig. 2c) and the percentage of rats showing preference toward the novel rat (score >0.55) was similar (Fig. 2d). However, in phase 2 of the test THC-exposed rats exhibited significantly impaired social memory, failing to distinguish between the novel *vs*. familiar rat (Fig. 2f) and displayed lower recognition *vs*. VEH and THC-NAC treated rats (Fig. 2g). Consistently, the percentage of animals preferring the novel rat was lower in the THC group (Fig. 2h). Thus, NAC prevented the deleterious impact of adolescent THC-exposure on social recognition memory.

### NAC prevents memory deficits induced by adolescent THC exposure

We first examined the effects of NAC on THC-induced impairments in the object recognition memory (NOR; Fig. 2i) task. During the acquisition phase both objects were explored similarly (paired *t*-tests for left *vs*. right object: p’s>0.05, *data not shown*) indicating no environmental bias. Following, one object was substituted and significant preference toward novelty was detected in all groups, except in THC exposed rats (Fig. 2j). This was reflected by significantly reduced object recognition scores in the THC group (Fig. 2k) and higher percentage of rats displaying no novelty preference (recognition score <0.55; Fig. 2m). Importantly, NAC supplementation reversed this deficit in THC exposed cohorts (Fig. 2k, m). Next, rats underwent the temporal order test (TMOR; Fig. 2n), which exploits rats’ preference to explore objects encountered earlier *vs*. more recently. During both acquisition phases, there were no side preferences observed (paired *t*-tests p’s > 0.05, *data not shown*). At the retention test, only THC exposed rats showed impaired recent object recognition (Fig. 2o), lower recognition score (Fig. 2p) and a higher percentage of rats with a recognition score <0.55 (Fig. 2r). Overall exploration times were homologous (Fig. 2q), confirming that the decreased temporal recall resulted from cognitive impairment and not from a reduced exploration. Finally, to determine if THC-induced memory deficits might be related to perceptual deficits, we employed the simultaneous oddity discrimination task (Supplemental Fig. 1a) which relies on the natural preference of rats to explore odd *vs*. similar objects. Analyses revealed no significant group differences across treatments in total exploration times or in oddity discrimination scores (Supplemental Fig. 1b, c), demonstrating that adolescent treatment with THC or NAC is not impacting perceptual capacity in adulthood. Summarizing, NAC prevented THC-induced object memory deficits without interfering with object recognition memory when applied alone.

### NAC prevents adolescent THC-induced impairments in cognitive flexibility

**Fig. 3:**
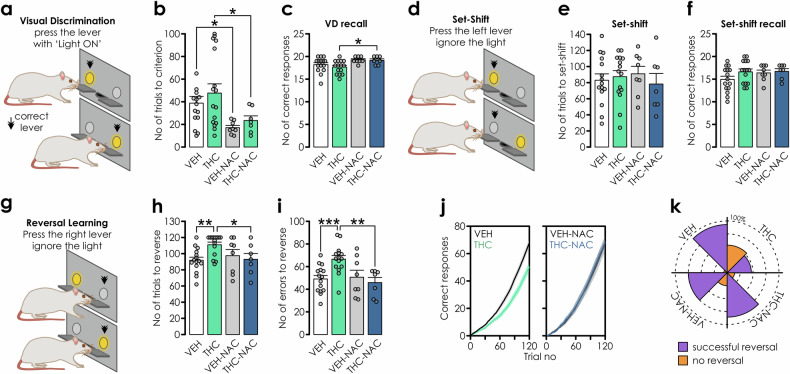
NAC prevents THC-induced cognitive flexibility impairments. **a,** **d,** **g** Schematics showing experimental design of the extra-dimensional set-shifting task. Rats were trained to press the lever above which a cue light was illuminated **a**. **b** NAC-treated rats acquired the association between pressing the illuminated lever and obtaining food reward faster than those drinking water (n: VEH = 14, THC = 16, VEH-NAC = 8; THC-NAC = 7). Two-way ANOVA: IP treatment: F_(1,41)_ = 1.057, p = 0.31; oral treatment: F_(1,41)_ = 9.682, p = 0.003; IP*oral treatment: F_(1,41)_ = 0.03, p = 0.863; pairwise comparisons: VEH *vs*. VEH-NAC: p = 0.04; THC *vs*. THC-NAC: p = 0.028. **c** Next day, the recall of this learned association was improved with NAC treatment *vs*. THC exposed groups: Two-way ANOVA: IP treatment: F_(1,41)_ = 0.551, p = 0.462; oral treatment: F_(1,41)_ = 8.571, p = 0.006; IP*oral treatment: F_(1,41)_ = 0.244; p = 0.624; pairwise comparisons: VEH *vs*. VEH-NAC: p = 0.086; THC *vs*. THC-NAC: p = 0.023. **d** Next, rats were required to press the lever on one side of the cage irrespective of the cue light. **e** The number of trials required to shift the strategy did not differ between groups. Two-way ANOVA: IP treatment: F_(1,41)_ = 0.21, p = 0.649; oral treatment: F_(1,41)_ = 0.001, p = 0.97; IP*oral treatment: F_(1,41)_ = 0.773, p = 0.384. **f** Set-shifting memory recall the following day was not affected by any treatment condition: Two-way ANOVA: IP treatment: F_(1,41)_ = 1.774, p = 0.19; oral treatment: F_(1,41)_ = 1.067, p = 0.308; IP*oral treatment: F_(1,41)_ = 0.777, p = 0.383. **g** However, changing the rule again to the opposite lever resulted in **h** increased numbers of trials required for the cognitive shift and **i** more errors committed by THC-exposed rats: Two-way ANOVA’s: #of trials: IP treatment: F_(1,41)_ = 2.037, p = 0.161; oral treatment: F_(1,41)_ = 1.426, p = 0.239; IP*oral treatment: F_(1,41)_ = 5.294, p = 0.027; pairwise comparisons: VEH *vs*. THC p = 0.002; THC *vs*. THC-NAC p = 0.02; # of errors: IP treatment: F_(1,41)_ = 2.284, p = 0.138; oral treatment: F_(1,41)_ = 5.177; p = 0.028; IP*oral treatment: F_(1,41)_ = 7.002, p = 0.011; pairwise comparisons: VEH *vs*. THC p < 0.001; THC *vs*. THC-NAC p = 0.001. **j** Average performance of rats during the reversal learning. Note the rightward shift in THC-exposed rats, reflecting more erroneous responses. This pathological error shift is prevented by NAC treatment. **k** Schematic summarizing the % of rats that successfully acquired the new cognitive strategy tactic. Note the profound deficit in THC treated cohorts and prevention in the NAC-treated cohorts.

Given the importance of the PFC in attentional and cognitive flexibility [47], we examined whether NAC would prevent THC-induced deficits in these domains. We used a set-shifting task [47] that assess attentional flexibility, modelled after the Wisconsin Card Sorting Test used in schizophrenia patients, who display impaired reversal learning in this task [48]. First, rats were trained to press a lever below an illuminated cue light for a food reward (Fig. 3a). Successful training occurred in all treatment groups with NAC treatment alone improving acquisition speed (Fig. 3b). Testing visual discrimination memory retrieval revealed no THC effects, while NAC co-treatment enhanced it (Fig. 3c), indicating memory persistence in all groups and intact consolidation processes. Next, rats were required to ignore the cue light and always press the same lever (set-shifting; Fig. 3d). There were no differences between groups and all groups switched to the new rule (Fig. 3e), retrieving the memory the following day (Fig. 3f). Changing the rule again (reversal learning, Fig. 3g), significantly increased the number of trials (Fig. 3h) and errors (Fig. 3i) selectively in THC-treated rats. As evident from the average cumulative correct responses across the session (Fig. 3j), learning in THC animals was delayed when compared to VEH rats. Moreover, the percentage of animals that did not reverse was elevated only in the THC cohort (Fig. 3k). Thus, adolescent antioxidant treatment with NAC is sufficient to prevent the deleterious side-effects of adolescent THC exposure on cognitive flexibility.

### Effects of adolescent THC exposure and NAC on the acoustic startle reflex, pre-pulse inhibition and startle habituation at adulthood

**Fig. 4:**
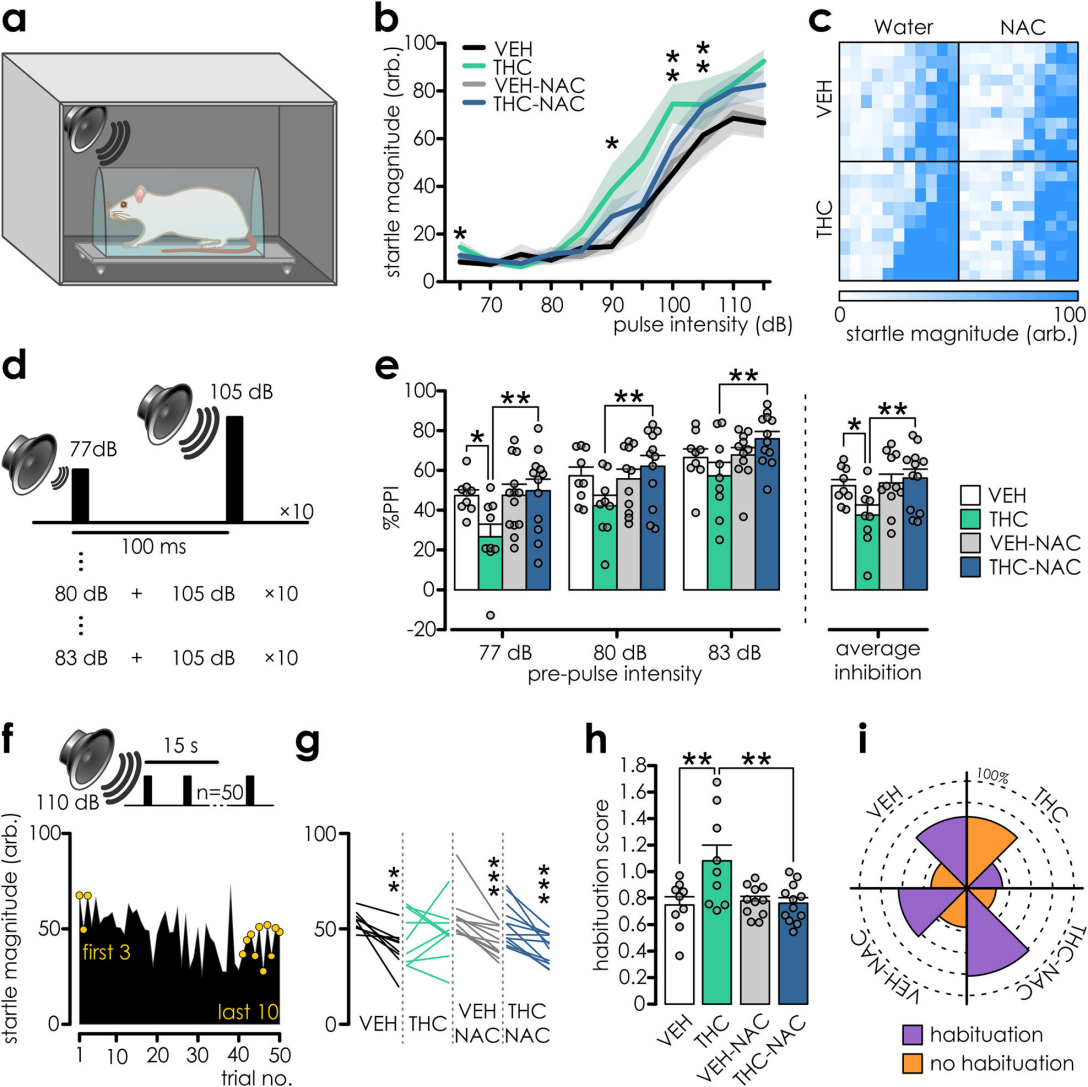
Adolescent THC-induced sensory filtering deficits are prevented by NAC. **a** Schematic representation of startle apparatus. **b** THC-exposed rats display increased startle reactivity (data presented as mean ± SEM; n: VEH = 9, THC = 9, VEH-NAC = 11; THC-NAC = 12). Two-way repeated measures ANOVA: Within-subjects effects: startle amplitude: F_(10, 370)_ = 121.994, p < 0.001; startle amplitude*IP treatment: F_(10, 370)_ = 2.514, p = 0.006; Startle amplitude*oral treatment: F_(10, 370)_ = 0.57, p = 0.838; startle amplitude*IP*oral treatment: F_(10, 370)_ = 0.609, p = 0.807; between-subjects effects: IP treatment: F_(1,37)_ = 0.002; oral treatment: F_(1,37)_ = 1.258; p = 0.269; IP*oral treatment: F_(1,37)_ = 1.379, p = 0.248; pairwise comparisons: pulse intensity: VEH *vs*. THC: 65 dB p = 0.026; 90 dB p = 0.025; 100 dB p = 0.007; 105 dB p = 0.009. **c** Depiction of startle response magnitudes across individual animals (rows within the squares) to startle pulse presentation of increasing intensity (discrete pixels; 65–115, 5 dB increment; lowest intensity at the leftmost position of the squares). The startle magnitude is color coded with inset below the graph. **d** Schematic depiction of PPI protocol. **e** Reduced PPI in THC-exposed rats was reversed with NAC. Two-way RM ANOVA: tests of within-subjects effects: startle inhibition: F_(2,74)_ = 50.099; p < 0.001; between-subjects effects: IP treatment: F_(1,37)_ = 1.2, p = 0.28; oral treatment: F_(1,37)_ = 5.698; p = 0.022; IP*oral treatment: F_(1,37)_ = 5.768; p = 0.021; pairwise comparisons for means: VEH *vs*. THC: p = 0.025; THC *vs*. THC-NAC: p = 0.002, pairwise comparisons for prepulse intensity: VEH *vs*. THC at 77 dB: p = 0.017, at 80 dB: p = 0.058, at 83 dB: p = 0.19; THC *vs*. THC-NAC: at 77 dB: p = 0.005; at 80 dB: p = 0.009; at 83 dB: p = 0.007. **f** Schematic of short-term startle habituation protocol and example depicting gradual decrease of startle responses. **g** Paired *t*-test revealed that median startle amplitude from last ten trials did not differ from first three trials in the THC group (VEH: t_(8)_ = 3.988, p = 0.004; THC: t_(8)_ = −0.329, p = 0.751; VEH-NAC: t_(10)_ = 4.897, p < 0.001; THC-NAC: t_(11)_ = 4.806, p < 0.001). **h** The habituation score remained high only in THC group. Two-way ANOVA: IP treatment: F_(1,37)_ = 5.586, p = 0.023; oral treatment: F_(1,37)_ = 4.684, p = 0.037; IP*oral treatment: F_(1,37)_ = 6.869, p = 0.013; pairwise comparisons: VEH *vs*. THC: p = 0.002; THC *vs*. THC-NAC: p = 0.002. **i** Radial plot capturing the inverted percentage of animals with impaired startle habituation in the THC group.

Adolescent THC exposure leads to sensorimotor gating impairments manifesting as disrupted prepulse inhibition (PPI) of startle, a cardinal endophenotype of schizophrenia [49]. Thus, we examined the potential effects of NAC to prevent these effects. Assessment of the acoustic startle response to increasing tone intensities showed exaggerated acoustic reactivity in THC treated rats (Fig. 4a–c). Next, we assessed sensorimotor gating by measuring PPI (Fig. 4d). NAC and THC-NAC rats showed PPI similar to VEH controls, whereas THC rats had reduced PPI across multiple prepulse intensities (Fig. 4e). Lastly, we tested short-term startle habituation (Fig. 4f). In contrast to all other groups, the final *vs*. initial startle responses of THC group did not significantly differ (Fig. 4g) and startle habituation levels were significantly lower compared to VEH and THC-NAC rats (Fig. 4h). Accordingly, the percentage of rats displaying at least a 15% decrease of startle amplitude was lower only in the THC group (Fig. 4i), demonstrating that NAC protects against the deleterious THC effects on adulthood sensorimotor gating.

### Adolescent THC-induced neuronal hyperexcitability in the mPFC is prevented by NAC

**Fig. 5:**
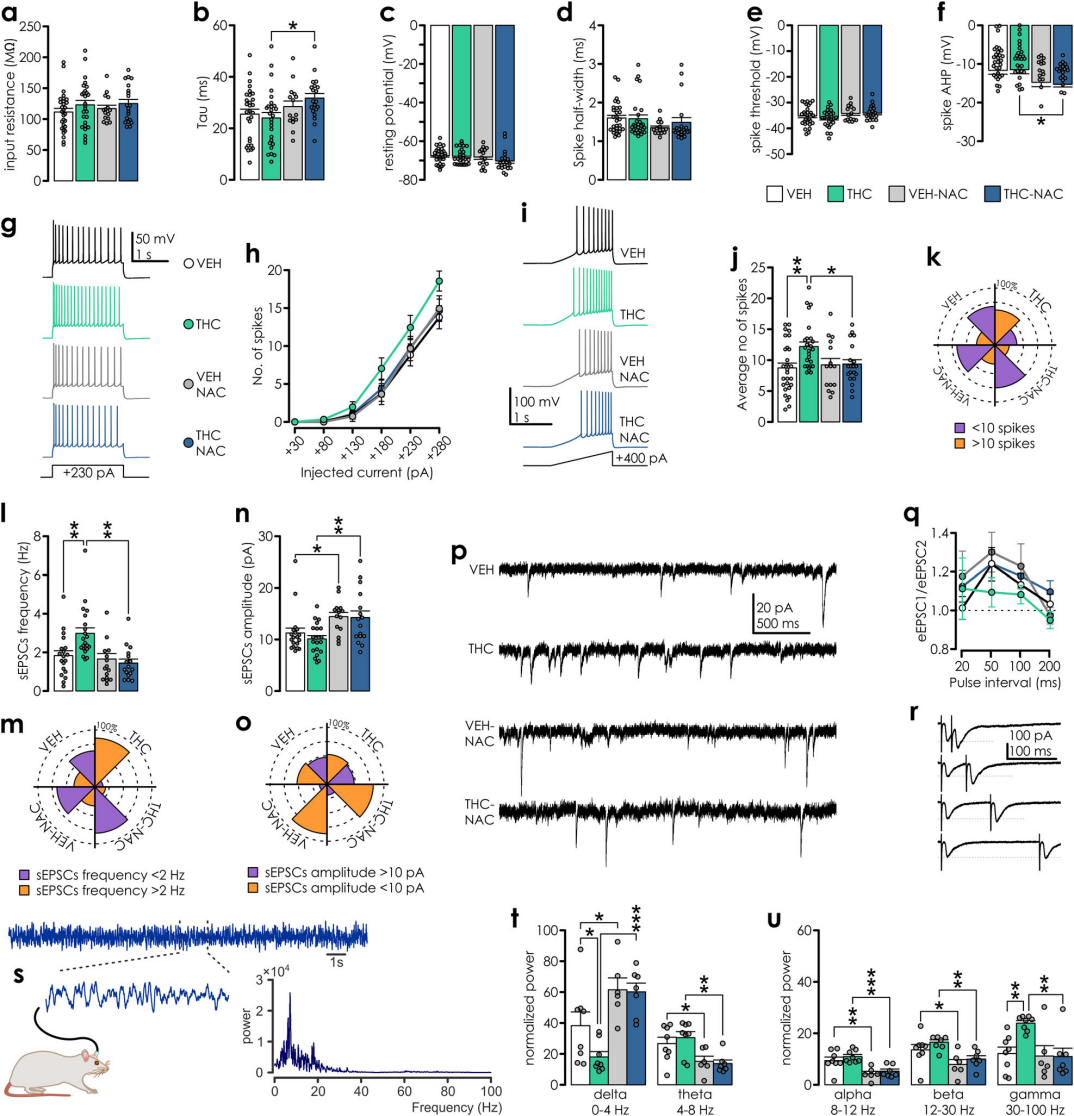
Adolescent THC-exposure-induced alterations in neuronal excitability and synaptic function are prevented by NAC co-treatment. **a** Input resistance, **b** membrane time constant and **c** resting membrane potential were not affected by adolescent THC treatment (n: VEH = 29, THC = 27, VEH-NAC = 15; THC-NAC = 20). Two-way ANOVAs: **a** IP treatment: F_(1,87)_ = 2.031,p = 0.158; oral treatment: F_(1,87)_ = 0.297, p = 0.587; IP*oral treatment: F_(1,87)_ = 0.064, p = 0.8. **b** IP treatment: F_(1,87)_ = 0.176, p = 0.676; oral treatment: F_(1,87)_ = 6.174, p = 0.015; IP*oral treatment: F_(1,87)_ = 1.252, p = 0.266; pairwise comparisons: THC *vs*. THC-NAC p = 0.01. **c** IP treatment: F_(1,87)_ = 1.477, p = 0.227; oral treatment: F_(1,87)_ = 2.586, p = 0.111; IP*oral treatment: F_(1,87)_ = 0.789, p = 0.377. **d** Spike half-width and **e** spike threshold also did not differ between groups. Two-way ANOVAs: **d** IP treatment: F_(1,87)_ = 0.335, p = 0.564; oral treatment: F_(1,87)_ = 3.288, p = 0.073; IP*oral treatment: F_(1,87)_ = 0.704, p = 0.404; **e** IP treatment: F_(1,87)_ = 0.009, p = 0.926; oral treatment: F_(1,87)_ = 3.075, p = 0.083; IP*oral treatment: F_(1,87)_ = 0.138, p = 0.711. **f** Spike AHP was augmented by NAC treatment. Two-way ANOVA: IP treatment: F_(1,87)_ = 0.023, p = 0.88; oral treatment: F_(1,87)_ = 10.52, p = 0.002; IP*oral treatment: F_(1,87)_ = 0.107, p = 0.745; pairwise comparisons: VEH *vs*. VEH-NAC p = 0.05; THC *vs*. THC-NAC p = 0.01. **g** Response of representative layer 2/3 mPFC pyramidal neurons obtained from different treatment groups to current step of +230 pA (square pulse illustrated below voltage trace). **h** Neuronal gain curves indicate a trend toward increase in excitability following THC exposure, although no interactions with treatments were detected. Two-way repeated measures ANOVA: depolarization step: F_(5,435)_ = 244.787, p < 0.001; depolarization step*IP treatment: F_(5,435)_ = 1.817, p = 0.108; depolarization step*oral treatment: F_(5,435)_ = 0.556, p = 0.734; depolarization step*IP*oral treatment: F_(5,435)_ = 1.526, p = 0.180. **i** Example traces illustrating action potential firing in response to a current ramp (ramping current injection from 0–400 pA in 1.5 s; 0.266 pA/ms). **j** Average no. of spikes/ramp was significantly increased following THC exposure. Two-way ANOVA: IP treatment: F_(1,85)_ = 5.197, p = 0.025; oral treatment: F_(1,85)_ = 2.251, p = 0.137; IP*oral treatment: F_(1,85)_ = 3.874, p = 0.052; pairwise comparisons: VEH *vs*. THC p = 0.001; THC *vs*. THC-NAC p = 0.013. **k** Percentage of cells with more/less than 10 spikes in a ramp across treatment groups. **l** sEPSCs frequency was increased in cells from THC-exposed rats (n = 15–21/group). Two-way ANOVA: IP treatment: F_(1,67)_ = 3.039, p = 0.086; oral treatment: F_(1,67)_ = 10.265, p = 0.002; IP*oral treatment: F_(1,67)_ = 6.475, p = 0.013; pairwise comparisons: VEH *vs*. THC p = 0.002; THC *vs*. THC-NAC p < 0.001. **m** Percentage of cells with sEPSC freq< or >2 Hz across different groups (n: VEH = 17, THC = 21, VEH-NAC = 15; THC-NAC = 16). **n** sEPSCs amplitude was increased in cells from NAC exposed rats, but not affected by THC exposure. Two-way ANOVA: IP treatment: F_(1,67)_ = 0.497, p = 0.483; oral treatment: F_(1,67)_ = 15.118, p < 0.001; IP*oral treatment: F_(1,67)_ = 0.269, p = 0.606; pairwise comparisons: VEH *vs*. VEH-NAC: p = 0.022; THC *vs*. THC-NAC: p = 0.002. **o** Percentage of cells with average sEPSCs amplitude smaller/larger than 10 mV across treatments. **p** Examples of sEPSCs recorded from adult pyramidal neurons following different adolescent treatments. **q** Paired-pulse facilitation ratio is not affected by adolescent treatment (n: VEH = 11, THC = 9, VEH-NAC = 15; THC-NAC = 17). Two-way RM ANOVA: stimulation interval: F_(3,144)_ = 6.805,p < 0.001; stimulation interval*IP treatment: F_(3,144)_ = 0.832, p = 0.478; stim interval*oral treatment: F_(3,144)_ = 0.172, p = 0.915; Stim interval*IP*oral treatment: F_(3,144)_ = 1.311, p = 0.273. **r** Example recordings representing EPSCs evoked by stimulation pulses delivered with 20, 50, 100 and 200 ms interval (from top to bottom). **s** Schematic depiction of LFP recording with an example raw signal trace and power spectrum analysis (inset). **t** and **u** Average normalized power for specific oscillatory bands was affected by adolescent treatments (n: VEH = 8, THC = 8, VEH-NAC = 6; THC-NAC = 7). Two-way ANOVAs for delta: IP treatment: F_(1.25)_ = 2.523, p = 0.125; oral treatment: F_(1.25)_ = 23.012, p < 0.001; IP*oral treatment: F_(1.25)_ = 1.919, p = 0.178; pairwise comparisons: VEH *vs*. THC: p = 0.035; VEH *vs*. VEH-NAC: p = 0.026; THC *vs*. THC-NAC: p < 0.001; theta: IP treatment: F_(1.25)_ = 0.109, p = 0.744; oral treatment: F_(1.25)_ = 15.214, p < 0.001; IP*oral treatment: F_(1.25)_ = 0.533, p = 0.472; pairwise comparisons: VEH *vs*. VEH-NAC: p = 0.038; THC *vs*. THC-NAC: p = 0.003; alpha: IP treatment: F_(1.25)_ = 1.428, p = 0.243; oral treatment: F_(1.25)_ = 25.1, p < 0.001; IP*oral treatment: F_(1.25)_ = 0.076, p = 0.785; pairwise comparisons: VEH *vs*. VEH-NAC: p = 0.003; THC *vs*. THC-NAC: p < 0.001; beta: IP treatment: F_(1.25)_ = 2.449, p = 0.13; oral treatment: F_(1.25)_ = 14.204, p < 0.001; IP*oral treatment: F_(1.25)_ = 0.083, p = 0.776; pairwise comparisons: VEH *vs*. VEH-NAC: p = 0.024; THC *vs*. THC-NAC: p = 0.007; gamma: IP treatment: F_(1.25)_ = 4.432, p = 0.045; oral treatment: F_(1.25)_ = 6.456, p = 0.018; IP*oral treatment: F_(1.25)_ = 4.784, p = 0.038; pairwise comparisons: VEH *vs*. THC: p = 0.004; THC *vs*. THC-NAC: p = 0.002. Patch clamp recordings were taken from layer 2/3 mPFC pyramidal neurons obtained from 28 adult male rats (VEH = 7, THC = 8, VEH-NAC = 5; THC-NAC = 8).

Adolescent THC exposure causes enduring mPFC neuronal abnormalities, including hyperactivity of pyramidal neurons in vivo and increased gamma power of oscillatory potentials [4, 5]. We next conducted ex vivo whole-cell patch-clamp recordings from layer 2/3 pyramidal neurons from the adult mPFC. THC treatment did not affect passive membrane properties relative to VEH (Fig. 5a–c), and action potential properties were similar across groups (Fig. 5d, e), except for enlarged spike afterhyperpolarization amplitude upon NAC treatment (Fig. 5f). There was a trend towards augmented neuronal gain in response to increasing depolarizing current injection in the THC group (Fig. 5g, h) and a significant increase of spiking upon a depolarizing ramp (Fig. 5i–k) along with a trend toward lower rheobase values (Supplemental Fig. 2). Furthermore, the frequency but not amplitude of spontaneous excitatory postsynaptic currents (sEPSCs) in THC rats was significantly higher indicating escalation of excitatory synaptic input. Although NAC prevented this frequency shift (Fig. 5l–p) it increased sEPSCs amplitude. Lastly, we measured presynaptic changes by employing paired-pulse ratio (PPR) and we detected no effects of adolescent treatments on evoked excitatory postsynaptic currents (ePSCSs; Fig. 5q, r).

To further explore the impact of excitability changes at the circuit level, we examined local field potentials (LFP) in freely behaving rats (Fig. 5s). Prior work has identified increased mPFC gamma power in anesthetized rats following adolescent THC exposure [5]. Consistently, we observed that adolescent THC exposure significantly increased gamma-power, concomitant with an impaired excitatory-inhibitory balance. Although the excessive THC-induced contribution of gamma band oscillations was reversed by antioxidant co-treatment, NAC also had significant effects on slower oscillatory bands, namely, by increasing delta and reducing theta, alpha and beta powers relative to VEH controls (Fig. 5t, u).

Together, these results demonstrate that adolescent THC exposure has no effect on passive membrane properties of PFC pyramidal cells, however, it significantly increases excitability in response to depolarization and spontaneous excitatory synaptic transmission, which further manifested in increased gamma power in behaving animals, consistent with previously reported hyperactivity of PFC neurons and dysregulation of γ-oscillations [4, 5]. Importantly, NAC treatment normalized both the frequency of EPSCs and γ-band power contributions.

### THC-induced molecular abnormalities in mPFC are prevented by NAC

**Fig. 6:**
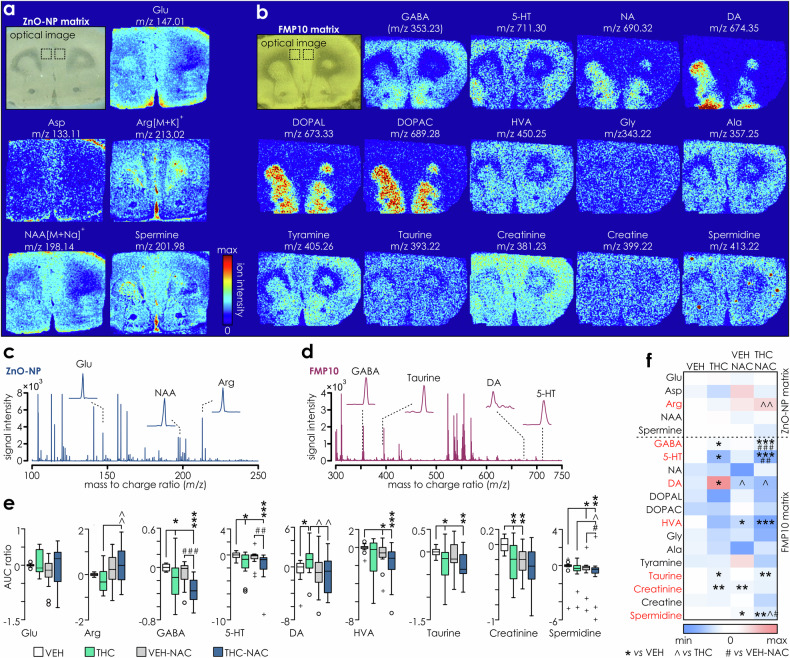
MALDI-MS quantification of amino-acids, neurotransmitters and their metabolites within mPFC indicates persistent changes induced by adolescent treatments. Optical images of coronal mPFC sections using ZnO-NP **a** or FMP-10 matrices **b**, followed by ion distribution plots for selected molecules. Metabolites were simultaneously assessed in a single scan for each matrix, and images are from a single VEH rat. The identity of visualized molecules and corresponding experimental *m/z* values are indicated above the plots. At each sampling position, 40 shots were used to acquire data in the *m/z* 50-500 (ZnO-NP) and *m/z* 299–1000 (FMP-10) range. Ion intensity data were visualized using a color scale 0–100% (inset on the right) except for 5-HT (0–80%); NA (0–75%); spermine (0–60%); DA, DOPAL, DOPAC, Arg, Asp (0–50%), Gln (0–30%) for best visualization. Lateral resolution, 100 µm. Average mass spectra from the mPFC region (delineated in optical images in **a** and **b**) facilitated by derivatization with ZnO-NP (blue; **c**) and FMP10 (crimson; **d**) with mass peaks for selected molecules displayed on insets. **e** Box plots of average log2 transformed ratios (ratio = AUC treatment/AUC VEH) for Glu, Arg, GABA, 5-HT, DA, HVA, taurine, creatinine and spermidine and **f** summary color plot of all assessed molecules showing the decrease (blue) or increase (pink) in the ion abundances across treatment groups relative to VEH (n = 16/group). Molecule identities are listed on the left side of the graph, where red colors indicate significant (p < 0.05) effect of statistical comparisons (2-way ANOVA followed with pairwise comparisons or Kruskal-Wallis test followed with Mann-Whitney tests). Significant post-hoc between-groups comparisons (*vs. VEH, ^ *vs*. THC, # *vs*. VEH-NAC) are indicated with: single (p < 0.05), double (p < 0.01) or triple symbol (p < 0.001) respectively. Arg (2-way ANOVA: IP treatment: F_(1,60)_ = 0.035, p = 0.853; oral treatment: F_(1,60)_ = 7.474, p = 0.008; IP*oral treatment: F_(1,60)_ = 1.368, p = 0.247; pairwise comparisons: THC-NAC *vs*. THC p = 0.008). GABA (Kruskal-Walis: H_(3)_ = 22.18, p < 0.001; THC *vs*. VEH: U = 64, p = 0.015, THC-NAC *vs*. VEH: U = 2, p < 0.001, THC-NAC *vs*. VEH-NAC: U = 40, p = 0.001). 5-HT (Kruskal-Walis: H_(3)_ = 13.05, p = 0.005; THC *vs*. VEH: U = 65, p = 0.017; THC-NAC *vs*. VEH: U = 42, p = 0.001, THC-NAC *vs*. VEH-NAC: U = 59, p = 0.008). DA (Kruskal-Walis H_(3)_ = 8.877, p = 0.031; THC *vs*. VEH: U = 62, p = 0.012, THC *vs*. VEH-NAC: U = 68, p = 0.023, THC *vs*. THC-NAC: U = 66, p = 0.019). HVA (K-W: H_(3)_ = 11.731, p = 0.008; VEH-NAC *vs*. VEH: U = 69, p = 0.026, THC-NAC *vs*. VEH: U = 32, p < 0.001). Taurine (K-W H_(3)_ = 9.612, p = 0.022; THC *vs*. VEH: U = 68, p = 0.023; THC-NAC *vs*. VEH: U = 58; p = 0.007). Creatinine (2-way ANOVA: IP treatment: F_(1,60)_ = 8.807, p = 0.004; oral treatment: F_(1,60)_ = 6.634, p = 0.012; IP*oral treatment: F_(1,60)_ = 2.075, p = 0.155; THC *vs*. VEH p = 0.003; VEH-NAC *vs*. VEH p = 0.006). Spermidine (K-W test H_(3)_ = 14.152, p = 0.003; VEH-NAC *vs*. VEH: U = 50, p = 0.009; THC-NAC *vs*. VEH: U = 29, p < 0.001; THC-NAC *vs*. THC: U = 68, p = 0.041; THC-NAC *vs*. VEH-NAC: U = 62, p = 0.037).

Previous studies reported that adolescent THC exposure leads to persistent changes in various molecular signalling pathways in the mPFC and associated neurotransmitter profiles associated with DA, GABA and Glu [4, 5]. Accordingly, we next used MALDI imaging (Fig. 6a, b) to measure, across treatment groups, the relative abundance of multiple neurotransmitters: glutamate (Glu), glycine (Gly), γ-amino-butyric acid (GABA), dopamine (DA), noradrenaline (NA) and serotonin (5-HT); their metabolites/precursors: 3,4-dihydroxyphenylacetaldehyde (DOPAL), 3,4-dihydroxyphenylacetic acid (DOPAC) and homovanillic acid (HVA); and neuroactive amino acids and molecules: aspartate (Asp), arginine (Arg), alanine (Ala), N-acetylaspartate (NAA), tyramine, taurine, creatinine, creatine, spermine and spermidine (Fig. 6c, d). The average levels (Fig. 6e, f & Supplemental Fig. 3) of Glu and NA remained unaffected by treatments, whereas GABA and 5-HT were significantly decreased in THC and THC-NAC groups (Fig. 6i, j). Amongst other metabolites, significant effects were observed for Arg (increased in THC-NAC treated *vs*. VEH); taurine (decreased in THC and THC-NAC treated groups), creatinine (decreased in THC and VEH-NAC treated groups *vs*. VEH) and spermidine (decreased in VEH-NAC and THC-NAC treated groups). Finally, DA was significantly increased in THC rats, but not in NAC and THC-NAC treated rats. Exposure to THC-NAC significantly reduced HVA levels (Fig. 6g, h, k, l).

## Discussion

With the rising prevalence of adolescent high potency cannabis use [50, 51], there is an urgent need to understand the neuropathophysiological events underlying the increased risk for schizophrenia and other neuropsychiatric disorders in adulthood. Here, we demonstrate that in male rats, THC-induced cognitive deficits including impaired sociability, recognition, and working memory impairments as well as cognitive flexibility and sensorimotor processing disturbances were mitigated by oral antioxidant NAC supplementation. Furthermore, THC-induced electrophysiological abnormalities in PFC neuronal excitability and synaptic transmission, aberrant oscillatory activity states and several altered neurochemical profiles were normalized by NAC. These novel findings emphasize the critical role played by THC-induced redox alterations and underscore the therapeutic potential of targeting oxidative stress-related pathophysiology to prevent cannabis-induced psychiatric risk mechanisms.

The endocannabinoid (eCB) system comprises a complex array of signaling molecules, associated synthesizing and degrading enzymes, transporters and receptors. The two main endocannabinoids, anandamide (AEA) and 2-arachidonoylglycerol (2-AG), are synthesized on demand from membrane lipids by associated lipases, and upon release bind with variable affinity to CB1 and CB2 receptors, peroxisome activated receptors (PPARs), the transient receptor potential cation channel subfamily V member 1 (TRPV1), the G-protein-coupled receptor 55 (GPR55) and serotonin 1 A receptors [52–54]. Importantly, the eCB system can also be targeted with phytocannabinoids, like THC. THC acts a partial CB1 receptor agonist, and other targets include CB2, glycine receptors, GPR55 and 5-HT3AR [54]. Activation of neuronal CB1 receptors attenuates synaptic transmission but may also increase extracellular glutamate concentrations [55], which might trigger mitochondrial impairment and oxidative stress [56]. AEA might induce apoptotic effects via TRPV1-mediated increase in intracellular Ca^2+^, mitochondrial uncoupling, and ROS formation [53, 57], while activation of PPAR-α by endocannabinoids dampens oxidative stress through upregulating the transcription of antioxidant enzymes [53, 57]. THC and AEA are also agonists for the GPR55 receptor, associated with anti-inflammatory and anti-oxidative functions by increasing levels of superoxide dismutase, glutathione (GSH) and catalase. Moreover, GPR55 might heterodimerize with CB1 receptors, aiding in the prevention of toxin-induced cell death [53, 58]. The interaction between the eCB system and redox homeostasis is bidirectional and occurs via multiple mechanisms, though mtCB1 plays a crucial role [52, 57]. CB1 receptor activation in macrophages is linked to promoting oxidative stress [53] but can reduce oxidative stress in the hippocampus [59], suggesting cell-type specific effects. In contrast, CB2 receptor activation is typically associated with lower ROS production [53].

Due to high energy demands, neurons are particularly vulnerable to oxidative stress [60], especially developing interneurons [61, 62]. Oxidative stress can cause cellular damage, consecutively altering neurotransmission and triggering cognitive impairments. Intracellular reactive oxygen species (ROS) side products of catalyzed reactions by ROS-generating enzymes, with NADPH oxidase (NOX) and nitric oxide synthase (NOS) being the main sources [63]. Antioxidative responses are triggered by activation of nuclear factor erythroid 2-related factor 2 (Nrf2) and ROS are neutralized with antioxidants, superoxide dismutases, glutamate-cysteine ligase and GSH [64]. Oxidative stress might affect eCB system, by increasing AEA and 2-AG levels, upregulation of CB1 and CB2 expression, and downregulation of FAAH [53]. The interaction between the eCB system and neurotransmission, redox balance and inflammation is complex and bidirectional, with each influencing the other in various physiological and pathological conditions [52, 57]. Importantly, THC exposure has been shown to alter glutathione levels in the brain [65], alter the expression of oxidative stress genes [66] and related proteins, inhibit mitochondrial respiratory rate [36], increase mitochondrial H_2_O_2_ production and free radical leak [40], and enhance brain metabolism and lipid peroxidation [67]. Moreover, THC-activated mitochondrial CB1Rs (mtCB1Rs) in astroglia decrease lactate release leading to increased redox stress in neurons and increased levels of mitochondrial ROS [32]. Therefore, the direct antioxidative properties of NAC and its effects on GSH synthesis provide a means to fight harmful oxidation. MtCB1Rs constitute ~15% of the total cellular CB1Rs, and ~30% of neuronal mitochondria contain CB1Rs, which directly regulate mitochondrial respiration and contribute to strong DSI [68]. They also modulate neuronal activity and synaptic release by regulating intracellular calcium and energy supply [69, 70]. In fact, mtCB1Rs facilitate mitochondrial calcium intake in astrocytes and mediate lateral potentiation of excitatory synaptic transmission [71], and their activation disrupts memory consolidation and retrieval [72]. For example, mtCB1Rs localized in astroglia and inhibitory terminals of hippocampal CA1 are strongly downregulated after acute THC exposure, while in cortical regions, only in excitatory terminals [73]. Thus, THC exposure can produce differential effects in regionally selective inhibitory/excitatory network dynamics known to be dysregulated in cognitive disorders. Importantly THC dose-dependently impairs mitochondrial function [40, 74] and can also conjugate with GSH and cysteine, the two crucial components of the redox system [75]. Therefore, THC might deplete internal GSH stores and intensify oxidative stress upon prolonged exposure, while NAC supplementation ensures a continuous supply of cysteine [76] and thus directly counteracts this effect.

With special relevance to the current study are previous investigations showing that developmental THC exposure downregulates gene expression for neurotrophic receptor *Trkb*, upregulates *Cb1r*, increases the *Nrf2/Keap1* ratio (indicating oxidative stress), and increases the pro-apoptotic marker BAX [77]. It also alters gene expression related to synaptic function, ion channels, mitochondrial biology and many psychiatric disease-associated genes [38], decreases NMDA currents, dampens glutamate receptor expression [78, 79], and reduces PFC GABA levels [80]. Further, THC upregulates cyclooxygenase-2 (COX-2) and elevates prostaglandin E_2_ production, indicative of inflammation [79]. Of note, COX-2 is suppressed by 2-AG in response to pro-inflammatory and excitotoxic insults [81] highlighting the divergent effects of THC *vs*. eCBs. Periadolescent THC exposure in mice disrupted protein expression of mitochondrial complexes I-IV, induced loss of membrane integrity and increased the load of mitochondrial proteins suggesting increased mitochondrial size and number, potentially to compensate for oxidative stress [82]. Importantly, mitochondrial impairment is linked with cognitive pathophysiology and can be ameliorated with antioxidants [83]. Consistent with our data, antioxidant treatments have proven to be effective in counteracting many pathological phenotypes associated with neurodevelopmental disorders [10, 41–43]. For example, NAC has been shown to reverse inhibitory and synaptic deficiencies in cortical interneurons differentiated from induced pluripotent stem cells of schizophrenia patients [84], prevent the development of cognitive impairments in multiple pre-clinical schizophrenia models [10, 85] and improve mismatch-negativity and clinical symptoms in schizophrenia patients [85–87]. Structurally, NAC serves as an antioxidant that undergoes deacetylation to cysteine required for GSH synthesis and therefore can boost GSH production and sustain cellular redox status [88]. Additionally, NAC may improve mitochondrial dysfunction through its positive impact on mitochondrial membrane potential and permeability [89], which are negatively affected by THC exposure [40, 82].

Interestingly, chronic THC exposure profoundly upregulates several neuroinflammatory markers, like cyclooxygenase-2 (COX-2) and prostaglandin E_2_ (PGE_2_) predominantly in the astroglia [79] and causes degeneration of cortical neurons via CB1R-mediated release of cytochrome c and activation of caspase-3 [90]. NAC also suppresses inflammatory cytokines and interferes with proinflammatory gene expression via inhibition of nuclear factor kappa-light-chain-enhancer (NF-κB) [88, 89] and thus may potentially counteract both THC-induced inflammation and oxidative stress. Finally, NAC can modulate Glu transmission via the astrocytic cystine/glutamate antiporter system x_C_^−^ [91]. In contrast, systemic administration of NAC does not impact Glu release patterns in PFC [92] and indeed, Glu levels measured by MALDI in the present study remained stable across groups. While future studies are required to explore these mechanisms, this suggests that the observed ameliorative effects of NAC in the context of adolescent THC exposure might be more functionally related to its antioxidant properties rather than direct glutamatergic modulation via system x_c_^−^.

Besides long-term schizophrenia-related deficits in behaviour and cognition, adolescent THC exposure induces many molecular and electrophysiological abnormalities, especially in the mPFC [4–7]. For example, in rodents, PFC layer 5 pyramidal neurons (PNs) display significant abnormalities following THC exposure, including more depolarized resting membrane potentials and lower spike thresholds [93]. This contrasts with the data presented here for layer2/3 PNs, where THC exposure did not alter resting membrane potentials or spike thresholds, but strongly facilitated neuronal excitability. However, similar to hippocampal phenotypes [79], THC exposure increased the frequency of EPSCs in PNs of layer 2/3 and layer 5 [93] suggesting that long-term adolescent THC impacts may diverge at the cellular level by differentially affecting neurons in specific cortical layers and brain regions but converge at the synaptic level by augmenting excitatory inputs. The EPSC frequency increase is largely attributable to presynaptic elements, suggesting overall network hyperactivity. Although the impact of an increased EPSC frequency at the functional level is not clear, it elevates background noise and therefore affects signal-to-noise ratio and potentially interferes with information processing, synaptic integration and various forms of plasticity [94]. Thus, elevated synaptic excitation following adolescent THC exposure could result, either individually, or collectively, from (i); synaptic imbalances caused by hyperactive mPFC glutamatergic neurons, (ii); diminished inhibition due to decreased GABA levels, or (iii); NMDA receptor hypofunction, leading to mPFC hyperexcitation via interneuron mediated disinhibition of pyramidal cells.

Interestingly, reduced cortical GABAergic drive is associated with a shift of γ-power toward higher frequencies and disinhibition of cortical neurons [95]. Here, we show that oscillatory PFC activity following THC exposure is characterized with lower δ and increased γ power, corroborating results from resting state electrophysiology findings in cannabis users and suggesting increased cortical activation [96]. Increased PFC gamma power specific to high frequencies (>60 Hz) may also be related to increased bursting rates of sub-cortical DA-VTA neurons [97], consistent with our previous evidence showing that adolescent THC treatment leads to persistent hyperactivation and increased bursting rates of DA cells [4, 5, 10]. This is also consistent with our MALDI PFC imaging results showing reduced GABA and increased DA expression. Activated postsynaptic CB1 receptors bind to NMDAR1 subunits and promote NMDAR internalization [98]. Thus, excessive CB1 activation with exogenous THC may lead to NMDAR hypofunction which in turn downregulates genes involved in synthesis, recycling and utilization of GSH in developing neurons, promoting oxidative stress. Of note, pharmacologically induced PFC NMDAR hypofunction leads to increased γ oscillations [99] while genetic deletion of NMDAR1 subunits in pyramidal neurons increases γ oscillations, cell excitability and sEPSC frequency [100], similar to the present data. Although NAC co-exposure was not able to compensate for THC-induced GABA reductions, it normalized relative DA content, spontaneous EPSCs frequency, gamma power and behavioural abnormalities, underscoring the relevance of excitation/inhibition balance and its sensitivity to oxidative stress in these phenomena.

Using MALDI, we performed an extensive analysis on the metabolomic mPFC landscape following adolescent THC exposure. We demonstrate a host of novel neurochemical abnormalities consistent with schizophrenia-like phenotypes and our observed cognitive and electrophysiological cortical alterations. While mPFC Glu levels remained unaffected, the GABA signal was significantly reduced in THC exposed animals, suggesting lower inhibitory drive in the mPFC network and a strong increase of EPSC frequency consistent with previous findings [4, 5]. Conversely, mPFC DA signal levels were significantly increased, and this effect was blocked by NAC. DA is a strong modulator of prefrontal activity and increases excitability of pyramidal cells in response to depolarization via D1 receptors [101]. PFC DA signaling also potentiates gamma oscillation power [102]. Hence, increased DA signaling might contribute to mPFC hyperexcitability as reported here and previously reported hyperactive DA neurons in the ventral tegmental area [4, 5]. Interestingly, DA may also directly inhibit mitochondrial respiration [103] and DA oxidation generates toxic quinones and ROS leading to mitochondrial dysfunction [104]. We also observed decreased mPFC taurine levels following THC exposure that was not prevented with NAC treatment. Taurine enhances GABA receptors and has neuroprotective functions against glutamatergic excitotoxicity. It elicits neuronal hyperpolarization via chloride channels suggesting that taurine deficiencies may lead to increased mPFC neuronal excitability. Therefore, decreased mPFC taurine levels might limit the defensive capabilities of mPFC neurons and NAC could potentially compensate for this loss by enhancing GSH synthesis and rebalancing the local redox system. In addition, we found that NAC treatment prevented THC-induced increases in the polyamine spermidine, which is known to regulate glutamatergic signaling by potentiating NMDA-receptor sensitivity to glycine [105]. Thus, NAC’s neuroprotective effects may involve its ability to reduce cortical spermidine levels and thereby limit local excitotoxic effects in the PFC during THC exposure, further indicated by our electrophysiological findings demonstrating a hyperactive neuronal mPFC phenotype. Finally, NAC/THC co-exposure significantly increased PFC arginine levels. Arginine is known to possess neuroprotective effects in inflammatory states by inhibiting HIF1α [106]. Furthermore, hyper-dopaminergic states induced by amphetamine can induce mitochondrial damage through the HIF1α pathway [107], suggesting another potential pathway by which NAC might serve a neuroprotective function against THC-induced cortical pathology.

### Study limitations

Oral NAC is associated with gastrointestinal discomfort, nausea, and diarrhea, but these symptoms are generally mild and occur at similar rates in placebo groups, indicating that NAC is well-tolerated and safe for chronic use [108]. In our study, we did not observe an increase in loose stools in NAC-treated animals during daily monitoring, consistent with previous research showing no toxicity after 30 days of high-dose NAC (1200 mg/kg/day) in rats [109]. After oral administration, NAC is primarily metabolized in the liver to cysteine, which enters the bloodstream [110]. Both NAC and cysteine have limited ability to cross the blood-brain barrier (BBB) [111]. Despite this, NAC administration increases GSH levels in rats [112, 113] and humans [114]. Transport across the BBB may be facilitated by sodium-dependent transport system [76] or converting cysteine to cystine, which is subsequently exchanged with intracellular glutamate via the cystine-glutamate antiporter, and reduced back to cysteine for GSH production [115, 116]. This mechanism supports the use of oral NAC as a defence against neuronal oxidative stress and maintaining redox balance.

Similar to other antioxidants, NAC may exhibit pro-oxidant actions, but these are generally limited to higher doses of 550–1500 mg/kg/day [117–119]. In the current study, NAC was dissolved in drinking water at a concentration of 900 mg/L. Given that an average rat weighing 200 g drinks approximately 40–50 ml/day [120], it would consume 36–45 mg of NAC daily, corresponding to 180–225 mg/kg/day. Importantly, pro-oxidant NAC effects were not observed at concentrations of 50 or 500 mg/kg making pro-oxidative actions of NAC in present study highly unlikely. Nonetheless, optimal doses, treatment schedules and testing efficacy of other antioxidants in preventing THC-induced abnormalities remain to be established. In addition, future studies should explore how NAC (and other antioxidants) may differentially protect the adolescent brain, comparing both female and male experimental cohorts.

The use of a single antioxidant compound in our study is a limitation, but previous research indicated that substituting NAC with other antioxidants can equally prevent behavioral deficits in neurodevelopmental model of schizophrenia [11]. Indeed, several compounds that counteract behavioral aberrations induced by adolescent THC exposure also exhibit antioxidant properties. For example, L-theanine has been shown to reduce oxidative stress by increasing total antioxidant capacity, GSH, and superoxide dismutase [121, 122] and to protect against DA-induced neurotoxicity via boosting GSH levels [123]. Similarly, cannabidiol (CBD), which also directly influences endocannabinoid transmission, diminishes ROS accumulation, activates the Nrf2 pathway through NF-kB inhibition and downregulates oxidative enzymes [124, 125]. CBD also directly influences eCB signalling by reducing CB1 receptor activation, promoting the inverse agonism of CB1 [126], and inhibiting eCB uptake and degradation [127–129]. Of note, CBD has shown behavioural benefits in rats with prenatal THC exposure [130] and in adult female rats with adolescent THC exposure [131]. Ebselen is another promising antioxidant that scavenges ROS and mimics glutathione peroxidase [132]. It has demonstrated neuroprotective effects [133–136] and mimicked NAC’s effects in a neurodevelopmental schizophrenia model [85], supporting the involvement of oxidative stress in the progression of cognitive impairments.

## Conclusions

Previous reports have linked THC exposure to mitochondrial and cellular alterations that ultimately result in oxidative stress. These neurodevelopmental insults may ultimately set up the brain for increased neuropsychiatric risk in later life. We report that treatment with a safe and well-tolerated oral antioxidant compound, NAC, can effectively prevent a host of adolescent-THC-induced pathophenotypes. Remarkably, these preventative effects were observable at the behavioural, electrophysiological and molecular levels of analysis, directly in the PFC. The effects were long-lasting and still present at early adulthood. Our pre-clinical findings provide rationale for the advancement of pharmacotherapeutic antioxidant treatments aimed at circumventing or perhaps reversing the deleterious effects of adolescent THC exposure.

## Supplementary information

Supplemental Materials

Supplemental Fig 1

Supplemental Fig 2

Supplemental Fig 3

## Data Availability

Further information and reasonable requests for resources and reagents should be directed to and will be fulfilled by the lead contact, Steven Laviolette (steven.laviolette@schulich.uwo.ca). The data reported in this paper will be shared by the lead contact upon reasonable request.
