# Supplementary material for: The antioxidant N-acetylcysteine prevents cortical neuropathological phenotypes caused by adolescent Δ-9-tetrahydrocannabinol exposure in male rats
Source: Transl Psychiatry. 2025 Oct 6;15:374. doi: 10.1038/s41398-025-03580-4 (PMC12501310; doi:10.1038/s41398-025-03580-4)
Supplement: Supplementary file 1 — Supplemental Materials [file 41398_2025_3580_MOESM1_ESM.docx]

# SUPPLEMENTARY MATERIALS & METHODS

### *SET-SHIFT AND REVERSAL LEARNING*

The set shifting procedure was performed as previously described^140^. Rats were food-restricted (15 g food/day) until they reached 85% of their initial body weight and habituated to a food reward (45 mg sucrose pellets; BioServ, USA). A sound attenuated box (Med-Associates, St Albans VT, USA) equipped with two levers, cue lights above the levers and food cup connected to a pellet dispenser and controlled with customized software procedures (MED-PC IV, Med-Associates) was used for testing.

First, rats were placed in the chamber (house light on, one lever extended) and learned the association between pressing the lever and food reward. When the lever was pressed 15 times it was retracted, and the other lever was inserted for 15 presses. Next, the levers were inserted randomly (15 times each) until pressed by the rat. Subsequently, the house light was switched off and scheduled presentation of levers started (trial duration 20s). Trials began with illumination of the chamber and insertion of one lever for 10 s. Upon a lever press the pellet was dispensed and the cue light remained on for 4 s. On failed trails, no food was dispensed, the lever was retracted after 10s and the house light was off until the end of trial. After 30 trials the rat was removed from the chamber. The next day the rat was exposed to same trial schedule until a performance of ≥85 presses/90 trials was achieved.

Subsequently the rats’ preferred lever was determined based on seven sets of trials (set: initial trial + secondary trial/s). Trials began with house lights on and insertion of both levers for 10s. After the rat pressed any lever, the food pellet was dispensed, and the light remained on for 4 s. In the following trial the rat needed to press the alternate lever to get the food. A set was considered complete when both levers were pressed. The lever with more presses on the initial trials was considered as ‘preferred’. Next day rats were exposed to 100 visual-cue discrimination (VD) trials. Upon trial start one of the cue light was illuminated and indicated the position of the to-be-rewarded lever. Three seconds later the chamber light was on and both levers inserted for 10 s. Only the correct press was rewarded. The learning session was considered successful when the rat pressed the correct lever 10 times in a row. The next day, to test the memory recall of the VD training, rats were presented with 20 VD trials. Subsequently, the response discrimination procedure started where the correct lever was set to the non-preferred lever irrespectively of the cue light position (min 120-max 150 trials). The successful set-shift was considered upon 8 successive correct responses. The total number of trials to set-shift and the total number of errors were calculated. On the last day, recall of the response discrimination memory was tested with 20 trials. Next, the correct lever was set to the alternate lever and reversal learning started (max 120 trials; performance criterion: 10 successive correct responses). The total numbers of trials to reverse and the total numbers of errors was calculated.

## *PATCH-CLAMP ELECTROPHYSIOLOGY*

Patch-clamp experiments were conducted as described previously^141,142^. Briefly, adult rats (PND75-PND130) with histories of adolescent THC *vs*. VEH treatments were anesthetised with isoflurane and decapitated (n=5-8 animals/group). Brains were briefly chilled in ice-cold saline, quickly extracted and cut in a coronal plane (300 μm) with a vibratome (Compresstome VF-200, Precisionary) while submerged in ice cold dissection solution (in mM: 2.5 KCl, 1.25 NaH_2_PO_4_-H_2_O, 24 NaHCO_3_, 10 MgSO_4_, 11 glucose, 234 sucrose, 2CaCl_2_, 3 myoinositol, 2 Na-pyruvate, and 0.4 ascorbate; equilibrated with 95% O_2_/5% CO_2_). Slices were transferred to a holding chamber containing artificial cerebro-spinal solution (ACSF) containing (in mM): 3 KCl, 1.25 NaH_2_PO_4_-H_2_O, 3 MgSO_4_, 26 NaHCO_3_, 124 NaCl, and 10 glucose; equilibrated with 95% O_2_/5% CO_2_ and subsequently 2 mM CaCl_2_ was added. Slices were incubated at 35°C for 30 min followed with 30 min at room temperature. Recording electrodes were borosilicate capillaries (1B150F-4, World Precision Instruments, Sarasota, FL) pulled on a horizontal P-97 puller (Sutter Instruments, Novato, CA) and filled with intracellular solution (in mM): 140 K-gluconate, 10 KCl, 1 MgCl_2_, 10 HEPES, 0.02 EGTA, 3 Mg-ATP, and 0.5 Na-GTP, pH adjusted to 7.3, 290-300 mosm/L. Recordings were conducted using Axon 700B amplifier and Digidata-1550 digitizer (Molecular Devices) acquired at 5 kHz and recorded on the computer for offline analysis using pCLAMP software (Molecular Devices; RRID:SCR_011323). Cells were recorded in whole-cell configuration and resting membrane potentials were measured directly after opening the cell. Subsequently, cells were held at -70mV and multiple protocols were executed in current-clamp mode to assess passive and active properties of the recorded cells. First, hyperpolarizing steps were applied (11 sweeps, from –400pA to + 0pA, 10s long, 40pA increments, step duration 2 s, interval 7s), following depolarizing steps (6 sweeps from +30pA to +180pA, 10s long, 30pA increments, step duration 2s, interval 7s) and ramp (4 sweeps, rise to +400pA, length 1.5s). Finally, we recorded spontaneous synaptic potentials in voltage-clamp mode for 5 min (holding potential –70mV). The following parameters were estimated from CC recordings: input resistance, time constant, capacitance, spike threshold (measured from the 1^st^action potential upon ramp depolarization), spike amplitude (peak potential−spike threshold), spike halfwidth (measured from the first spike using depolarizing steps), afterhyperpolarization amplitude (spike threshold−spike through), hyperpolarization-activated potential (measured as the difference between the sag and steady response potential to hyperpolarizing step evoking response closest to –80mV), slow afterhyperpolarization (sAHP=holding potential–minimum potential reached following the ramp protocol), number of spikes/depolarizing step of given intensity, number of spikes/ramp and rheobase (minimal current required to evoke an action potential estimated from ramp protocol).

## *MATRIX ASSISTED LASER DESORPTION/IONIZATION MASS SPECTROMETRY (MALDI-MS)*

Rats were given an overdose of pentobarbital (Euthanyl; 270 mg/ml), and brains were removed and flash frozen (–80°C). For quantification of multiple neurotransmitters and metabolites two different matrices were used, zinc oxide nanoparticles (ZnO-NP, Sigma-Aldrich, St. Louis, MO) and 4-(Anthracen-9-yl)-2-fluoro-1-methylpyridin-1-ium iodide (FMP-10, TAG-ON, Uppsala, Sweden; Cat#T1001) and are detailed in the Supplementary Materials and Methods. Frozen brains were cryo-sectioned (Leica Biosystems) in coronal plane (thickness 10 or 12 µm for ZnO-NP or FMP-10 respectively) and thaw-mounted onto conductive indium tin oxide-coated glass slides (Hudson Surface Technology, Old Tappan, NJ) and stored at –80°C. Each slide contained sections from all treatment groups. On the scanning day, sections were desiccated at room temperature and sprayed with ZnO-NP (1 mg/ml in 50% ACN) or FMP-10 (1.8 mg/ml in 70% ACN) matrix using a TM sprayer (HTX Technologies, Chapel Hill, NC). ZnO-NP was first filtered with a 1.2 μm filtered syringe and sprayed onto tissue sections at the following conditions: temperature, 65°C; flow rate, 0.05 ml/min; nozzle velocity, 1200 mm/min; track spacing, 3 mm; pressure, 10 psi; no of passes, 32. FMP-10 was sprayed onto the tissue sections at the following optimized conditions: temperature, 82°C; flow rate, 0.08 ml/min; nozzle velocity, 1100 mm/min; track spacing, 2 mm; pressure, 6 psi; no of passes, 30. As a calibration standard, 0.3 µL of 6.0 mg/mL α-cyano-4-hydroxycinnamic acid (CHCA, Sigma-Aldrich, St. Louis, MO) solution in ACN/H2O/TFA (50/50/0.1%, vol) was spotted near the tissue sections on each slide. Following the slides were put in a MALDI slide adapter and scanned using Sciex 5800 MALDI TOF/TOF mass spectrometer (Framingham, MA) equipped with a 349nm Nd: YLF laser (pulse rate 400 Hz) and images were acquired with Sciex TOF-TOF Series Explorer and TOF-TOF Imaging For MS. Mass spectra were acquired from a *m/z* range of 50 to 500 Da with ZnO-NP and a *m/z* range of 300-1000 Da with FMP-10. Spectra were collected by firing 400 shots/pixel. The laser energy was optimized prior to each run based on a balance between peak resolution and S/N. Lateral resolution was 70 µm in positive ion mode. MSIReader (1.02, North Carolina State University) was used for data visualization and average intensity for mPFC region was exported for further analysis in MatLab (The MathWorks, Inc.; RRID:SCR_011622) using custom-written scripts. Accurate mass was used for identification of putative neurotransmitters and metabolites corresponding to: Glu (*m/z* 147), Asp (*m/z* 133), Arg[M+K]^+^ (*m/z* 213), N-acetylaspartate [M+Na]^+^ (*m/z* 198), spermine (*m/z* 201), GABA (*m/z* 353.00), 5-HT (*m/z* 711.20), NA (*m/z* 690.25), DA (*m/z* 674.28), DOPAL (*m/z* 673), DOPAC (*m/z* 689.25), HVA (*m/z* 450.17), Gly (*m/z* 343), Ala (*m/z* 357.15), tyramine (*m/z* 405.20), taurine (*m/z* 393.13), creatinine (*m/z* 381.17), creatine (*m/z* 399.18), spermidine (*m/z* 413.27) as described previously (see Supplemental Table 1). Statistical analysis was performed on log2 transformed ratios calculated for area under the curve (AUC) for a given molecule using the formula: ratio=molecule AUC_treatment_/average molecule AUC_VEH_.
