# Supplementary figures and images for: The antioxidant N-acetylcysteine prevents cortical neuropathological phenotypes caused by adolescent Δ-9-tetrahydrocannabinol exposure in male rats
Source: Transl Psychiatry. 2025 Oct 6;15:374. doi: 10.1038/s41398-025-03580-4 (PMC12501310; doi:10.1038/s41398-025-03580-4)

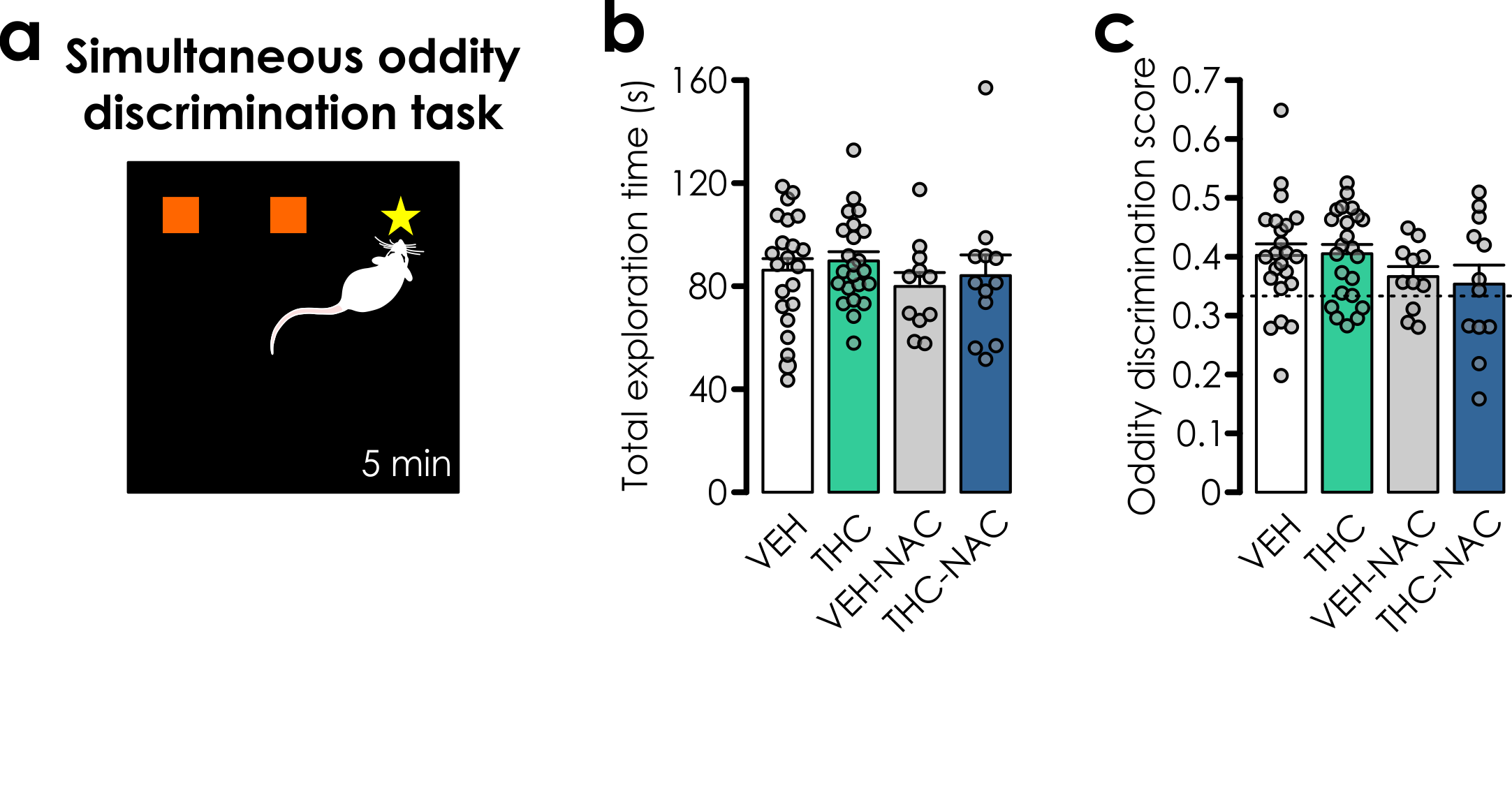

Supplement: Supplementary file 2 — Supplemental Fig 1 [file 41398_2025_3580_MOESM2_ESM.tif]

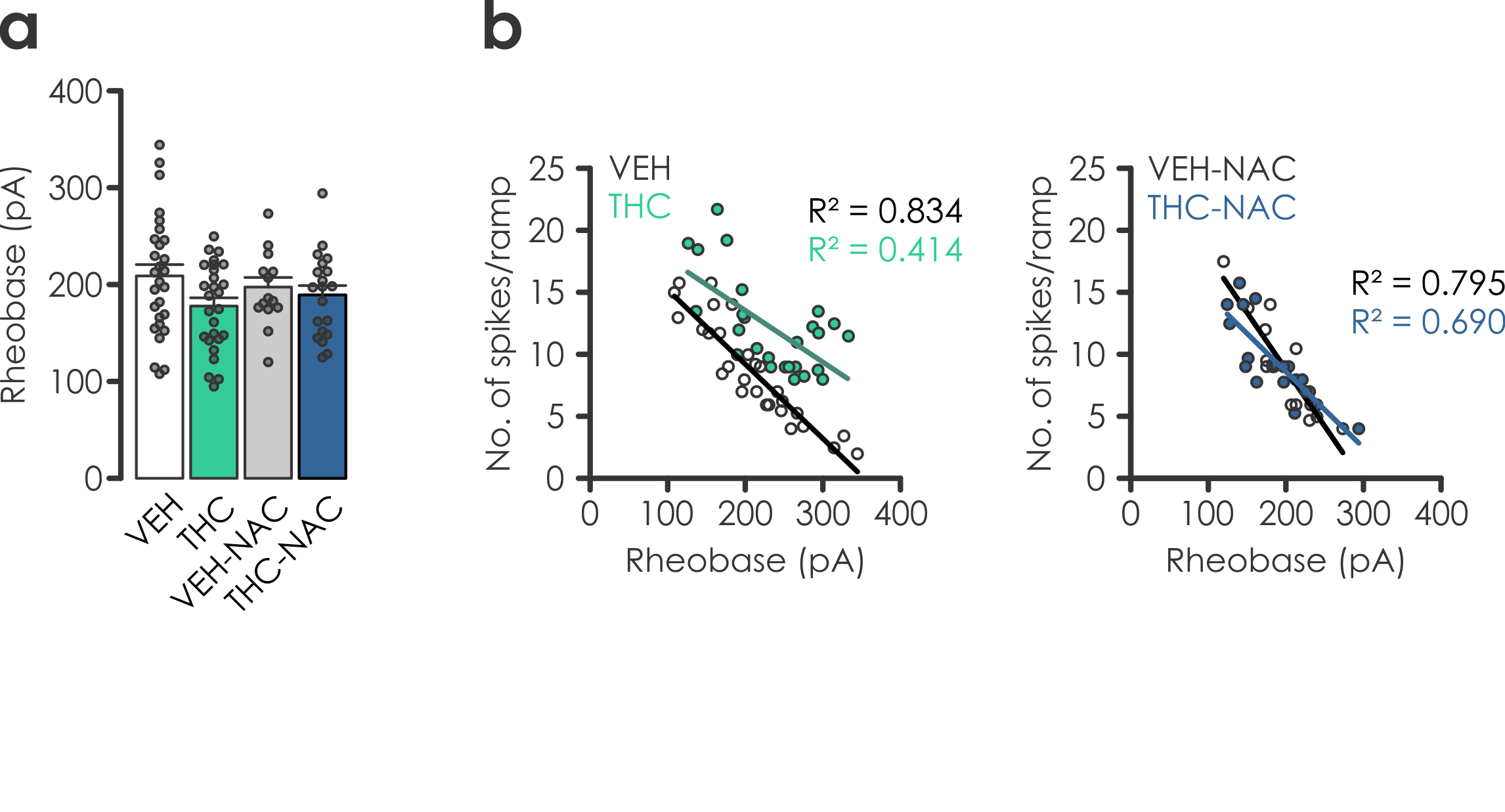

Supplement: Supplementary file 3 — Supplemental Fig 2 [file 41398_2025_3580_MOESM3_ESM.tif]

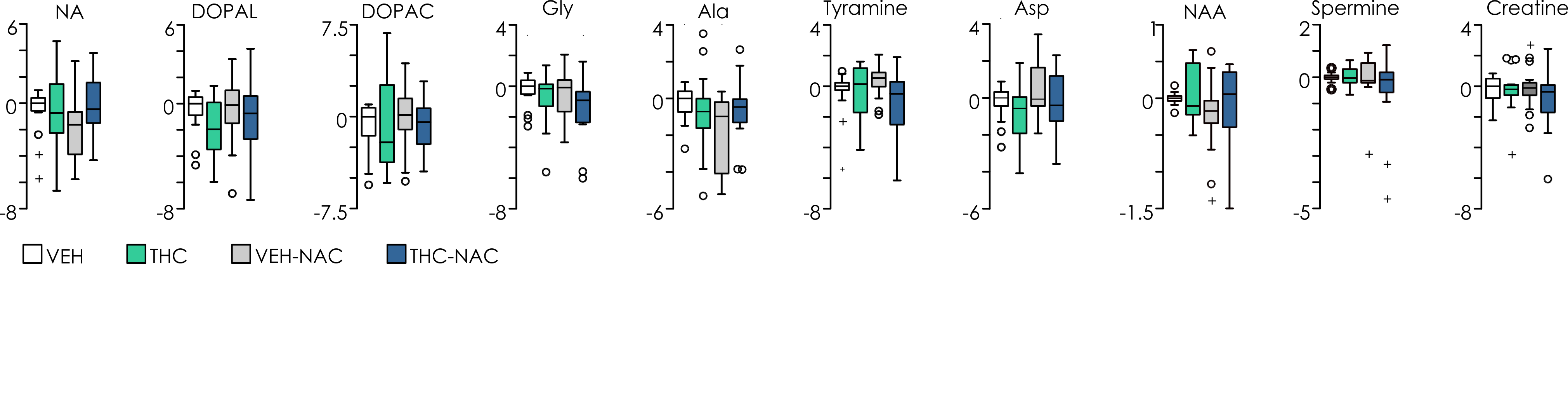

Supplement: Supplementary file 4 — Supplemental Fig 3 [file 41398_2025_3580_MOESM4_ESM.tif]
